# Influence of fecal fermentation on the anthelmintic activity of proanthocyanidins and ellagitannins against human intestinal nematodes and *Caenorhabditis elegans*

**DOI:** 10.3389/fphar.2024.1390500

**Published:** 2024-07-22

**Authors:** Jonathan Jato, Emmanuel Orman, Yaw Duah Boakye, François Ngnodandi Belga, Dieudonné Ndjonka, Emelia Oppong Bekoe, Eva Liebau, Verena Spiegler, Andreas Hensel, Christian Agyare

**Affiliations:** ^1^ Department of Pharmacognosy, School of Pharmacy, University of Health and Allied Sciences, Ho, Ghana; ^2^ Institute of Pharmaceutical Biology and Phytochemistry, University of Münster, Münster, Germany; ^3^ Department of Pharmaceutics, Faculty of Pharmacy and Pharmaceutical Sciences, Kwame Nkrumah University of Science and Technology, Kumasi, Ghana; ^4^ Department of Pharmaceutical Chemistry, School of Pharmacy, University of Health and Allied Sciences, Ho, Ghana; ^5^ Department of Biological Sciences, Faculty of Sciences, University of Ngaoundéré, Ngaoundéré, Cameroon; ^6^ Department of Pharmacognosy and Herbal Medicine, School of Pharmacy, College of Health Science, University of Ghana, Accra, Ghana; ^7^ Institute of Integrative Cell Biology and Physiology, University of Münster, Münster, Germany

**Keywords:** fecal fermentation, geraniin, procyanidin C1, human intestinal parasites, *C. elegans*, condensed and hydrolyzable tannins, *Phyllanthus urinaria*, *Combretum mucronatum*

## Abstract

Some tannin-rich plants such as *Combretum mucronatum* and *Phyllanthus urinaria* are widely used in Africa for the control of parasitic nematodes in both humans and livestock. Tannins have been recognized as an alternative source of anthelmintic therapies, and hence, recent studies have focused on both the hydrolyzable and condensed tannins. These groups of compounds, however, have poor oral bioavailability and are metabolized by gut microbiota into lower molecular weight compounds. The role of these metabolites in the anthelmintic activities of tannins has not been explored yet. This study investigated the effects of fecal metabolism on the anthelmintic potential of procyanidin C1 (PC1) and geraniin and the tannin-enriched extracts of *C. mucronatum* (CML) and *P. urinaria* (PUH), which contain these compounds, respectively. Metabolites were formed by anaerobic fermentation of the test compounds and extracts in a fresh human fecal suspension for 0 h, 4 h, and 24 h. Lyophilized samples were tested *in vitro* against hookworm larvae and whipworm (*Trichuris trichiura*) larvae obtained from naturally infected human populations in Pru West District, Bono East Region, Ghana, and against the wildtype strain of *Caenorhabditis elegans* (L4)*.* Both extracts and compounds in the undegraded state exhibited concentration-dependent inhibition of the three nematodes*.* Their activity, however, significantly decreased upon fecal metabolism. Without fermentation, the proanthocyanidin-rich CML extract was lethal against hookworm L3 (LC_50_ = 343.5 μg/mL, 95% confidence interval (CI) = 267.5–445.4)*, T. trichiura* L1 (LC_50_ = 230.1 μg/mL, CI = 198.9–271.2), and *C. elegans* (LC_50_ = 1468.1 μg/mL, CI = 990.3–1946.5). PUH, from which the ellagitannin geraniin was isolated, exhibited anthelmintic effects in the unfermented form with LC_50_ of 300.8 μg/mL (CI = 245.1–374.8) against hookworm L3 and LC_50_ of 331.6 μg/mL (CI = 290.3–382.5) against *T. trichiura* L1, but it showed no significant activity against *C. elegans* L4 larvae at the tested concentrations. Similarly, both compounds, procyanidin C1 and geraniin, lost their activity when metabolized in fecal matter. The activity of geraniin at a concentration of 170 μg/mL against *C. elegans* significantly declined from 30.4% ± 1.8% to 14.5% ± 1.5% when metabolized for 4 h, whereas that of PC1 decreased from 32.4% ± 2.3% to 8.9% ± 0.9% with similar treatment. There was no significant difference between the anthelmintic actions of metabolites from the structurally different tannin groups. The outcome of this study revealed that the intact bulky structure of tannins (hydrolyzable or condensed) may be required for their anthelmintic action. The fermented products from the gut may not directly contribute toward the inhibition of the larvae of soil-transmitted helminths.

## 1 Introduction

Globally, parasitic nematodes remain a significant threat to human and animal health, with a huge impact in tropical regions, where sanitation and hygienic practices are poor (Charlier et al., 2015; WHO, 2022). Currently, a limited range of synthetic drugs are being used as chemo-preventive therapies or treatment for worm infestations in humans and animals. The emergence of and widespread parasite resistance to anthelmintic drugs in livestock presents a significant threat to the future of anthelmintics in human health (Keiser and Utzinger, 2008; Williams et al., 2014a; Rose et al., 2020). Coupled with drug resistance is the limited number of drugs that are unavailable in certain jurisdictions. To achieve the World Health Organization’s 2030 targets for soil-transmitted helminthiases, there is a need for new and effective medicines to combat drug resistance and increase the access to treatment regimens in endemic areas (WHO, 2020).

This need for new drugs against gastrointestinal nematodes (GINs) has increased the interest in research into medicinal plants and their bioactive products as alternative sources of natural anthelmintic agents (Geary, 2012; Williams et al., 2014a; Hoste et al., 2015). Plants present this potential because they have, since ancient times, contributed to the discovery of many drug molecules and have served as remedies for primary healthcare, particularly in Africa (Agyare et al., 2009). Many medicinal plants from Africa have been reported to have promising potential as anthelmintic agents (Santos et al., 2019; Jato et al., 2022). In veterinary practices, orally administered plant extracts and animal feed prepared from tannin-rich plants have been investigated for the control of helminth infections (Min et al., 2003; Max et al., 2007).

Plant polyphenols, particularly tannins, are well-recognized for their potential as nematicidal agents, and many reports have recently focused on this group as bioactive compounds of interests (Santos et al., 2019; WHO, 2020; Jato et al., 2022). Due to their astringency, tannins appear to act through multiple pathways and mechanisms, targeting various stages of the parasite lifecycle (Spiegler et al., 2017; Greiffer et al., 2022). Some studies have also reported that simple, low-molecular-weight phenolic compounds such as gallic acid, ellagic acid, or gentisic acid, which often form the building blocks for tannins, may show anthelmintic activity (Ndjonka et al., 2013). What remains unclear is whether tannins act only in their unchanged state or do their gut metabolites play any role in their *in vivo* action in the gastrointestinal tract (Spiegler et al., 2017).

The microbiota of the human gut is known to play an important role in the metabolism and excretion of orally administered drugs (Piwowarski et al., 2016), and it can significantly influence the bioavailability and bioactivity of some phenolic molecules (Wiseman et al., 2004; Blaut and Clavel, 2007; Marín et al., 2015). When ingested, many plant constituents are fermented by the gut microflora into smaller molecules before being absorbed (Ito et al., 2008). Depending on their chemical nature and stability, some molecules may pass the stomach unchanged and are only degraded in the small intestine or colon (Ilett et al., 1990). Although some of the products of this metabolism are excreted through feces, others become the active drug constituents, which may either be absorbed or act locally in the intestines. In the case of GIN, active metabolites must preferably remain unabsorbed to achieve sufficient concentrations against parasites in the gut.

Proanthocyanidins (PAC), also referred to as condensed tannins (CT), are often poorly absorbed in the gut and pass through the stomach and small intestines unchanged (Kahle et al., 2007; Zumdick et al., 2012; Tao et al., 2019) and are then metabolized extensively in the colon. They are often subject to pH-dependent transformations into various phenolic metabolites, most of which have been credited for the *in vivo* therapeutic benefits of PAC (Ludwig et al., 2015). Although only small amounts of dimeric procyanidins, for instance, are reported to be absorbed intact (Holt et al., 2002; Sano et al., 2003), most are degraded in the colon into benzoic, hippuric, phenylacetic, phenylpropionic, and phenylvaleric acids, which may contribute to the therapeutic role of these polyphenols (Gonthier et al., 2003a; Gonthier et al., 2003b; Rios et al., 2003; Ou and Gu, 2014; Ludwig et al., 2015).

When administered orally, the ellagitannin (ET) subclass of hydrolyzable tannins also pass the stomach unchanged and are degraded by the microbiome of the colon into dibenzopyran-6-one derivatives often referred to as urolithins (Cerdá et al., 2003; Cerdá et al., 2004; Cerdá et al., 2005; Seeram et al., 2006). Urolithins can achieve relevant systemic concentrations and may be responsible for the pharmacological actions of ellagitannin-rich plants (García-Villalba et al., 2022; Banc et al., 2023). Some biological activities and therapeutic roles of ET and urolithins have been investigated (Cerdá et al., 2004; Espín et al., 2013).

*Phyllanthus urinaria* L (Phyllanthaceae) is locally known in Ghana as ‘Bɔwomaguw’akyi’ and is used in the treatment of many disease conditions, including intestinal parasites, malaria, enteritis, and hepatitis (Satyan et al., 1995; Agyare et al., 2014). It is also used in the management of dropsy, wounds, hypertension, jaundice, and asthma (Bharali et al., 2003; Hout et al., 2006; Lans, 2006). Extracts of *P. urinaria* and its major component, geraniin, have previously been shown to possess anthelmintic effects against various organisms, including *C. elegans, Trichuris suis, Ascaris suum, Ancylostoma caninum*, *Toxocara canis, Haemonchus contortus*, and *Trichostrongylus colubriformis* (Agyare et al., 2014; Spiegler, 2016; Karonen et al., 2020; Jato et al., 2023). Aqueous acetone extract of *P. urinaria* produced LC_50_ of 2.6 mg/mL against *C. elegans* and inhibited larval migration of *T. suis, A. suum, A. caninum*, and *T. canis* with IC_50_ values of 24.3 μg/mL, 35.7 μg/mL, 112.8 μg/mL, and 1,513.2 μg/mL, respectively. Geraniin also inhibited *C. elegans* larvae with an LC_50_ value of 2.5 mM (Jato et al., 2023).

*Combretum mucronatum* Schumach. and Thonn (Combretaceae) is a traditional remedy in Africa for treating malaria, diabetes, wounds, dysentery, guinea worm, hookworm, and pinworm infestations (Koné et al., 2012; Agyare et al., 2014; Kisseih, 2014). Extracts and oligomeric procyanidins (including procyanidin C1) from the leaves of *C. mucronatum* have also been reported to exhibit profound activity in various organisms, including *C. elegans* and some animal parasites (Koné et al., 2012; Spiegler et al., 2015; Herrmann and Spiegler, 2019; Greiffer et al., 2022). The ethanolic extract of *C. mucronatum* leaves induced 85.3% reduction of *Trichuris muris* worm burden in mice (Koné et al., 2012) and yielded an LC_50_ value of 1.67 mg/mL against *C. elegans* larvae (Spiegler et al., 2015), whereas procyanidin C1 induced a 47.3% inhibition of *C. elegans* larvae at 1 mM concentration (Spiegler, 2020).

This study investigated the effects of fecal fermentation on the anthelmintic activity of two polyphenolic compounds, geraniin and proanthocyanidin C1, and extracts containing these tannins against two human intestinal nematodes and the wildtype strain of the versatile free-living worm *C. elegans.*

## 2 Materials and methods

### 2.1 Ethical approval

The study with biological material from human volunteers was performed according to the relevant ethical guidelines. The study was approved by the Ethical Committee of Westfalen-Lippe, University of Münster, Germany (acceptance number 2017–526-f-S), in cooperation with the Committee on Human Research, Publication, and Ethics of Kwame Nkrumah University of Science and Technology, Kumasi, Ghana, with protocol numbers CHRPE/AP/532/19 and CHRPE/AP/331/20.

### 2.2 General experimental procedures

Except where stated, all solvents and reagents were procured from VWR (Darmstadt, Germany). Water was obtained using a Millipore Simplicity 185 system (Schwalbach, Germany). Extracts were prepared and compounds were isolated using standard procedures, as previously reported by Jato et al. (2023) and summarized below.

Column chromatography: medium-pressure chromatography on Sephadex LH-20 (Cytiva, Sweden); MCI Gel CHP-20P (75–150 μm, 450 Å) (Sigma-Aldrich Corp., St. Louis, USA).

Preparative HPLC system (Waters, Milford, MA, USA): 2545 Quaternary Gradient Module, prep degasser, 2998 PDA Detector, 2707 autosampler, and fraction collector III controlled by Waters ChromScope v1.40 Beta software. Stationary phase: RP 18 VP 250/21 Nucleodur C18 Htec (Macherey-Nagel, Düren, Germany); load: 10 mg/mL; flow rate: 15 mL/min; injection volume: max. 2 mL; and fraction size: 15 mL. Mobile phase gradient were A (0.1% formic acid in water) and B (0.1% formic acid in acetonitrile). Peaks were collected based on the UV max plot chromatogram (λ 200–400 nm).

Analytical UHPLC was carried out using an Acquity™ UPLC system with an RP-18 HSS T3 (2.1 mm × 100 mm, 1.8 µm) column, QD mass-selective detector (-and + scan, mass 100.00–1000.00 Da), and PDA λe detector (λ 210–400 nm), autosampler, in-line degasser run by Empower 3 software application (Waters), Flow rate: 500 μL/min, injection volume: 2–5 μL, and column temperature: 40°C. The eluting binary gradient mobile phase comprised 0.1% v/v formic acid in water (A) and 0.1% v/v formic acid in acetonitrile (B), where t_0min_: 2% B, t_2min_: 8% B, t_11min_: 18% B, t_15min_: 35% B, t_20–22min_: 100% B, and t_23–25min_: 2% B.

UPLC/+ESI-QqTOF-MS analysis was performed as previously described by Jato et al. (2023) using a UHPLC Dionex Ultimate 3000 RS Liquid Chromatography System: Dionex Acclaim RSLC 120 C18 column (2.1 × 100 mm I.D., 2.2 μm), detection: Dionex Ultimate DAD-3000 RS (λ 200–400 nm) and Bruker Daltonics micrOTOF-QII (Bruker, Bremen, Germany), ionization source: Apollo electrospray (positive mode), flow rate: 400 μL/min, injection volume: 2 μL, and binary gradient mobile phase: water with 0.1% formic acid (A) and acetonitrile with 0.1% formic acid (B), elution, where t_0min_: 5% B, t_0.4min_: 5% B, t_9.9min_: 100% B, t_15min_: 100% B, t_15.1min_: 5% B, and t_20min_: 5% B.

The isolation process was monitored by thin-layer chromatography (TLC) on silica gel 60 F_254_ precoated aluminum plates (Merck KGaA, Darmstadt, Germany). Resolution was carried out in the ethyl acetate/formic acid/water (90:5:5 *v*/*v*/*v*) mobile phase. The plates were derivatized with either 1% FeCl_3_, anisaldehyde/H_2_SO_4_ reagent with heating, natural product reagent A (1% diphenylboryloxyethylamine) with polyethylene glycol (PEG) 400, or vanillin/HCl reagent with heating. Detection was carried out using CAMAG TLC Visualizer II (visionCATS software) at wavelengths of 254 nm, 366 nm, and white light.

### 2.3 Preparation of plant extracts

An acetone–water (7:3 v/v) extract of *P. urinaria* L. was prepared by rotor–stator extraction (Ultra-Turrax T25 Digital, IKA Labortechnik, Staufen, Germany), as described previously by Jato et al. (2023).

Leaves of *C. mucronatum* were collected in January 2021 in Ngaoundere, Cameroon (north latitude of 7°85′88.57″ and east longitude of 13°59′39.19″). The sample was identified by a botanist, Prof Tchoupou Tsouala, at the University of Maroua, Cameroon, and a voucher specimen was deposited at the National Herbarium in Yaoundé, Cameroon, under the code number 32193 HNC. The remaining plant material was washed with water and shade-dried at room temperature. Powdered leaves were first defatted by soxhlet extraction with petroleum ether and subsequently extracted by rotor–stator using ethanol–water (1:1 *v*/*v*) solution in the manner as described for the *P. urinaria* extract (Jato et al., 2023). Detailed analytical characterization of the *P. urinaria* extract was performed as recently described by Orman et al. (2023).

**FIGURE 1:**
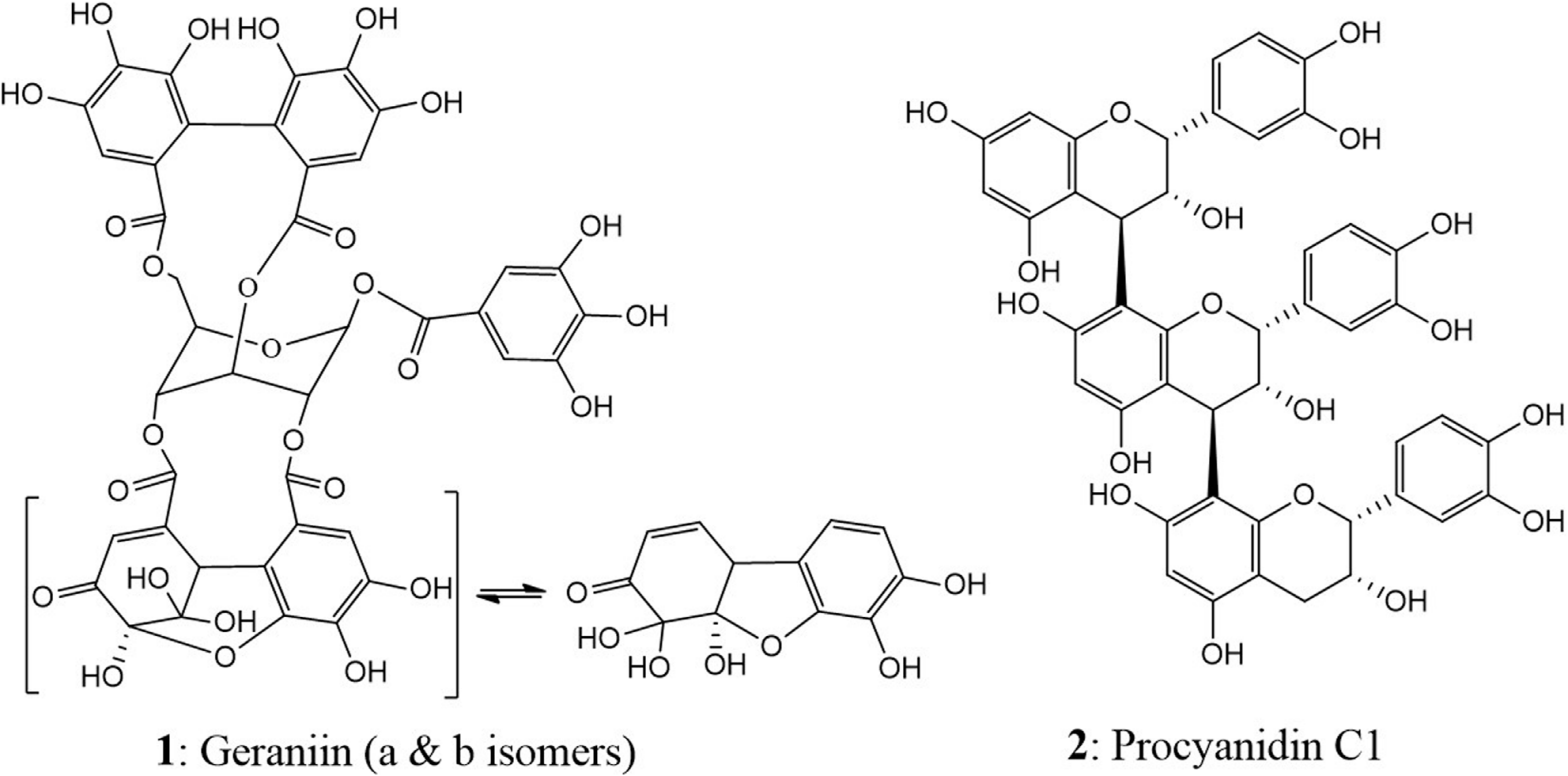
Chemical structures of geraniin (**1**) and procyanidin C1 (**2**).

Two polyphenolic compounds, geraniin (**1**), an ET, and procyanidin C1, representing condensed tannin (CT) (Figure 1), were used in the study. Geraniin was isolated from the hydro-acetonic extract of *P. urinaria* (Jato et al., 2023). Due to the high occurrence in the plant, procyanidin C1 (**2**) was isolated from the ethyl acetate fraction of the flowers of *Tilia platyphyllos* SCOP (Malvaceae), which were kindly donated by Dr. Nico Symma, University of Münster, Germany (IPBP 519) (Symma et al., 2020). This step helped in saving time and obtaining the compound in a good yield for the subsequent studies.

### 2.4 Isolation of procyanidin C1 (PC1) from *T. platyphyllos* ethyl acetate fraction

**FIGURE 2:**
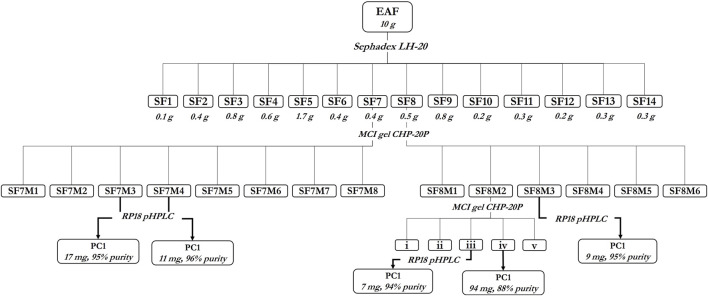
Isolation of procyanidin C1 (PC1) from the ethyl acetate fraction of *Tilia platyphyllos* Scop. flowers.

Proanthocyanidin C1 was isolated from the ethyl acetate fraction (EAF) of an aqueous extract from the flowers of *T. platyphyllos* through several chromatographic steps outlined in Figure 2.

In brief, 10 g of EAF was first fractionated on the Sephadex LH-20 stationary phase (isocratic 100% ethanol) to afford 14 fractions (SF1–SF14) (Supplementary Table 1). Fractions SF7 (0.4 g) and SF8 (0.5 g) were fractionated on MCI-Gel CHP-20P (gradient MeOH/H_2_O 1:20 →1:0) to yield SF7M1–SF7M8 and SF8M1–SF8M6 (Supplementary Table 2), respectively. Fraction SF8M2 (288 mg) was purified on the MCI-Gel CHP-20P stationary phase (MeOH/H_2_O 1:20 →1:0), yielding 94 mg of PC1. Fraction ‘iii’ of SF8M2 (25 mg), SF7M3 (198 mg), SF7M4 (59 mg), and SF8M3 (18.5 mg) were purified by preparative HPLC to yield 7 mg, 17 mg, 11 mg, and 9 mg of PC1, respectively, amounting to a total of 138 mg of PC1 (Figure 2).

The compound was identified by LC–MS analysis, as described above, in comparison to previous reports (Bicker et al., 2009; Kisseih et al., 2015; Spiegler et al., 2015) and a reference procyanidin C1 (in house, >99% by HPLC).

### 2.5 Fecal fermentation of tannins and tannin-containing extracts

*Ex vivo* human fecal fermentation of the test samples was performed at 0, 4, and 24 h time points, and the fermentation product was recovered for biological assays. Anaerobic buffer was prepared by dissolving L-cysteine HCl (0.015% w/v) in sterile M9 buffer (containing 22 µM KH_2_PO_4_, 42 µM Na_2_HPO_4_, 85.6 µM NaCl, and 1 µM MgSO_4_.7H_2_O) in 250-mL media bottles (Schott AG, Germany). The bottles were capped with re-sealable rubber stoppers, and an anaerobic condition was created by vacuum and bubbling with N_2_ gas for 30 min. This buffer solution was stored at 4°C and used for the subsequent work.

Test solutions were prepared by dissolving the extracts and compounds in anoxic M9 buffer in sealed vials to produce 10 mL stock solutions. The final concentrations were 0.17 mg/mL and 2 mg/mL for the extracts and 0.17 mg/mL for the compounds (178.5 µM and 196.2 µM for geraniin and procyanidin C1, respectively).

A fresh fecal sample was collected in a tightly sealed plastic sample container from a healthy, 31-year-old, male volunteer who was on a normal diet, a non-smoker, did not drink alcohol, and had not used antibiotics for the past 3 years. Approximately 60 g of the fecal sample was immediately transferred into a 1-L sterile media bottle, which was sealed and filled with nitrogen gas. The anoxic M9 buffer was then added to the fecal sample and stirred to achieve a 10% w/v human fecal suspension (HFS) using a magnetic stirrer. A measure of 27 mL of the HFS was injected into 100-mL pre-vacuumed stoppered media bottles, and 3 mL of the stock test solutions was added to achieve the respective final concentrations. An untreated control (UC) was prepared by adding 3 mL anoxic M9 buffer to 27 mL HFS. The set-up was incubated at 37°C in a G24 environmental incubator shaker (New Brunswick Scientific Co. Inc., USA) at 100 rpm for 4 h and 24 h.

After incubation (except the sample for 0 h), the samples were immediately cooled on ice, transferred into cold 50-mL Eppendorf tubes, and centrifuged at 3,500 *g* for 10 min at 4°C. The supernatant was again centrifuged at 20,000 *g* for 10 min, filtered through 0.45-µm nylon membrane filters (Wicom Perfect-Flow^®^), and lyophilized (Alpha 1-4 LD plus Christ). Each experiment was performed in triplicates, and three independent assays were conducted on separate days using fresh fecal samples and a time interval of at least 48 h.

### 2.6 Human intestinal parasites for *ex vivo* studies

The study involving human biological material was performed according to the relevant guidelines. Parasite eggs were obtained from volunteers from Adjalaja-Beposo, a rural community in the Pru West District of the Bono East Region of Ghana, in June 2022.

The participants were selected randomly using the convenience sampling (door-to-door) approach in households. The members of the selected households were educated on the exercise, and their consent was sought before each was given a set of labeled clean plastic sample containers. A demonstration was carried out on how to collect and transport the samples to the Community Health and Planning Services (CHPS) facility, where a mobile laboratory was set up for their processing.

The participants returned to the health facility in the mornings with freshly collected fecal samples in tightly capped sample containers. The samples were labeled and examined coproscopically using the Kato–Katz thick smear technique (Katz et al., 1972). Each sample was mounted on two microscope slides and independently examined by two scientists under the microscope for the presence of parasite ova using the World Health Organization (WHO) bench aids (Ash et al., 1994; Knopp et al., 2009). Egg-positive samples were identified, immediately stored in a cold ice-chest, and used for the egg isolation or coproculture method.

#### 2.6.1 Hatching of hookworms from coprocultures

Hookworm-positive samples were mixed with a 1:2 portion of activated charcoal and transported to the Kumasi Center for Collaborative Research (KCCR), Kwame Nkrumah University of Science and Technology, Kumasi, Ghana, where they were transferred into plastic Petri dishes and incubated in the dark at room temperature for 8 days.

The hookworm larvae were then harvested by a modified Baermann funnel method (Knopp et al., 2008). In brief, the incubated samples were transferred into cell strainers placed in a plastic sieve whose mesh was submerged in tap water in a funnel with a plastic hose and release clamp. The setup was covered with dampened tissue paper and incubated at room temperature for 24 h.

The first 50 mL containing the larvae was collected into centrifuge tubes and allowed to stand for 1 h. The top 40 mL supernatant was decanted, and the larvae were washed twice with tap water. The volume was reduced to 10 mL, and the larval population was determined by counting 10 × 10 µL of the larval suspension under the microscope. The larvae were stored in Hanks’ balanced salt solution (HBSS) medium; preserved with penicillin (10^4^ u/mL), amikacin (10 mg/mL), and amphotericin B (0.25 mg/mL); incubated at 27°C; and used for the assay.

#### 2.6.2 Hatching of *T. trichiura* larvae

Eggs of the whipworm were isolated from pooled feces of *T. trichiura-*positive individuals by the standard flotation–sedimentation techniques (Wimmersberger et al., 2013; Jato et al., 2023). In brief, the fecal sample was homogenized with an immersion blender and filtered through a 1-mm-mesh ordinary kitchen sieve. The flow-through was serially filtered in a stack of three 1-mm mesh sieves lined with different layers of gauze. The filtrate from the last sieve, which contained the eggs, was then pooled in a 1-L beaker and purified first by replacing the supernatant with 1 L saturated saline solution (1.1 specific gravity). This was vigorously shaken and left to stand for approximately 1 h. A measure of 10 mL of the egg sediment was added to 40 mL of the biphasic solution of saturated saline and 50% w/v sugar in 50-mL falcon tubes and centrifuged at 260 *g* for 5 min. The floating egg layer was pipetted and washed in a calico sieve with tap water. The sedimentation–flotation process was repeated until the eggs were free of any fecal matter. The ova were quantified and incubated in tap water at 25°C for 6 weeks until over 90% embryonation was achieved (Burden and Hammet, 1976).

The ova were hatched according to the method recently described for *T. suis* (Jato et al., 2023)*.* The L1 larvae were cleansed by migration through gauze and stored for a maximum of another 24 h before they were used for each test (Wimmersberger et al., 2013).

#### 2.6.3 Anthelmintic activity testing in human intestinal parasites

The test samples comprised the fecal metabolized lyophilizate of geraniin (GER), procyanidin C1 (PC1), extracts of *P. urinaria* aerial parts (PUH) and *C. mucronatum* leaves (CML), and an untreated control (UC). Each vial of the fecal lyophilizate of the test samples was reconstituted in a specified volume of deionized sterile water to achieve the respective stock concentrations similar to the starting concentration of the extracts or compounds. These were serially diluted to achieve test concentrations of 125, 250, 500, and 1,000 μg/mL for the extracts and 21.25, 42.5, 85, and 170 μg/mL for the compounds.

Anthelmintic activity was evaluated by pipetting 285 µL of the test solutions into 24-well plates, and 15 µL of the larval suspension (approximately 15 larvae) was added to each well. The plates were incubated at 37°C, and the larvicidal activity was evaluated after 72 h for hookworm and 24 h for whipworm. A binary scale of ‘dead or alive’ was used to assess the effects of the treatment. All larvae that were straight, immotile, or unresponsive upon addition of 50 µL warm water (70°C) to the wells were considered dead (Wimmersberger et al., 2013). Each concentration was tested in triplicates, and independent assays (using larvae from three different pools of feces harvested from different donors) were performed. The untreated control was made of the lyophilizate of the untreated sample from fecal fermentation, whereas albendazole (12.5–100 µM) was employed as the positive control.

#### 2.6.4 Anthelmintic activity against *C. elegans*

The *in vitro* activity of the fecal metabolites was additionally evaluated against *C. elegans* larvae*.* The wild-type strain (N2 Bristol) was obtained from the *Caenorhabditis* Genetics Center and cultured on Nematode Growth Medium using *Escherichia coli* OP50 strain as the food source (Stiernagle, 2006).

Synchronous L4 larvae were then tested against a gradient concentration of the test solutions in 24-well microtiter plates. The stock solutions were serially diluted with M9 buffer to achieve concentrations of 250, 500, 1,000, and 2,000 μg/mL for the extracts and 21.25, 42.5, 85, and 170 μg/mL for the compounds. A solution of the UC reconstituted in deionized sterile water served as the negative control for each time point, and 40 mM levamisole HCl (Glentham Life Sciences) was used as the positive control. Mortality was recorded after 72 h using an inverted light microscope, and worms were considered dead if they were straight, immotile, and unresponsive to eyelash probes. Each concentration was tested in three wells and in three biological replicates.

### 2.7 Statistical analysis

The raw data were recorded and transformed into percentage mortality using Microsoft Excel. This was exported to GraphPad Prism Ver.8.3.0 (GraphPad Software Inc.), and a non-linear regression analysis was performed. The best-fit values for LC_50_ of *C. elegans* and human intestinal nematodes were determined, and the mean mortality was compared using one-way ANOVA, followed by Dunnett’s test for multiple comparison. *p* < 0.05 were considered statistically significant for the test samples.

## 3 Results

The metabolites were produced by anaerobic fermentation of the compounds and extracts in fresh human fecal suspension for different durations. Although the characterization of these metabolites was not part of this study, there was enough spectroscopic evidence of the fermentation process. UPLC analysis of the fermented product showed a complete disappearance of peaks corresponding to the relevant phenolic compounds (geraniin and procyanidin C1). The lyophilizate of the fermentation product was tested against hookworm and whipworm larvae obtained from naturally infected human populations in Ghana and against the stage-4 (L4) larvae of *C. elegans*.

### 3.1 Anthelmintic effects on human intestinal parasites and *Caenorhabditis elegans*

**FIGURE 3:**
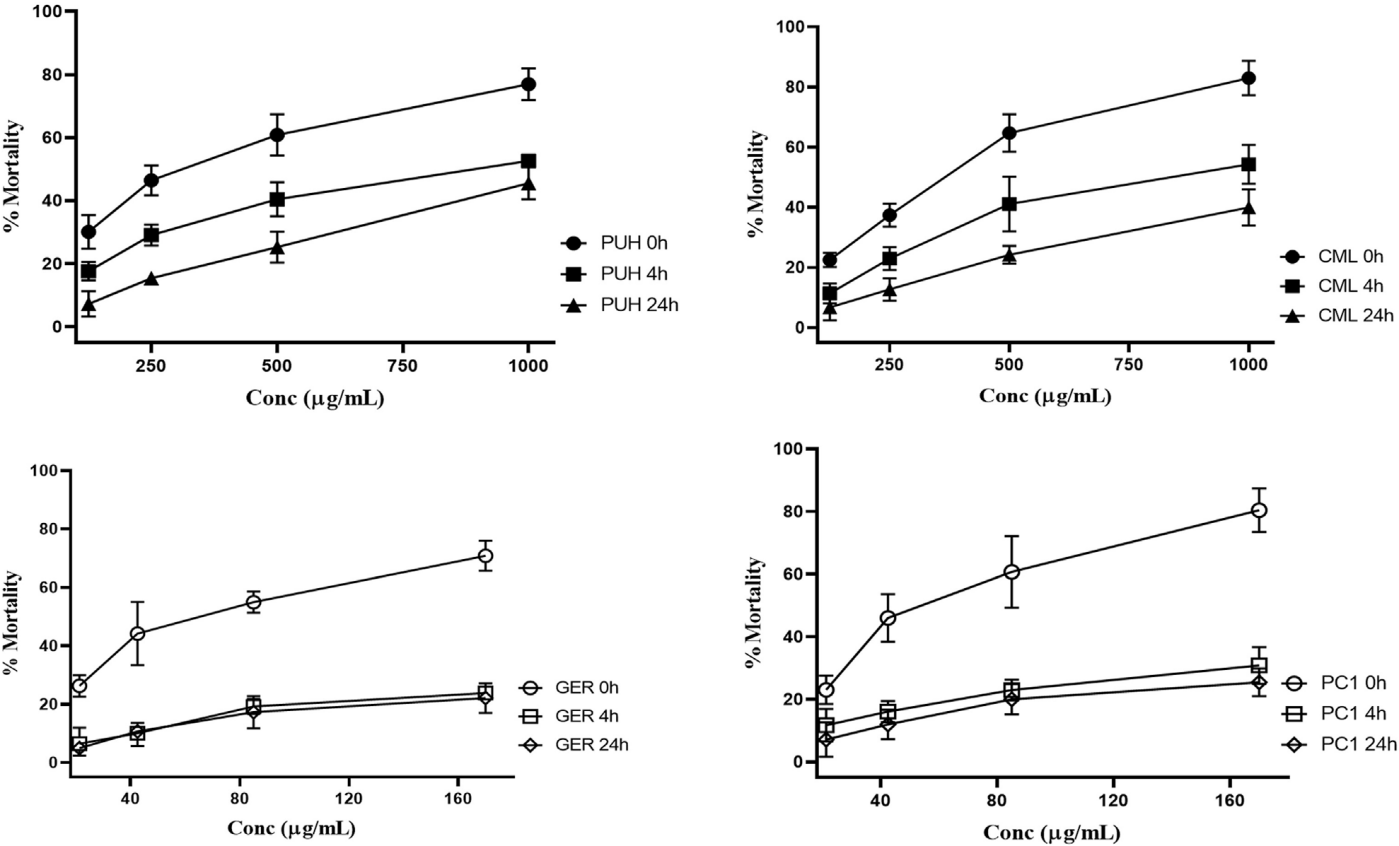
Relative anthelmintic activity of PUH **(A)**, CML **(B)**, ellagitannin geraniin **(C)**, and procyanidin C1 **(D)** without and after 4 and 24 h fermentation with human fecal suspension against hookworm larvae L3 after 72 h incubation at 37°C. PUH, *Phyllanthus urinaria* extract; CML, *C. mucronatum* extract; GER, geraniin; PC1, procyanidin C1. Bars represent mean ± SEM from three independent experiments with three technical replicates.

**FIGURE 4:**
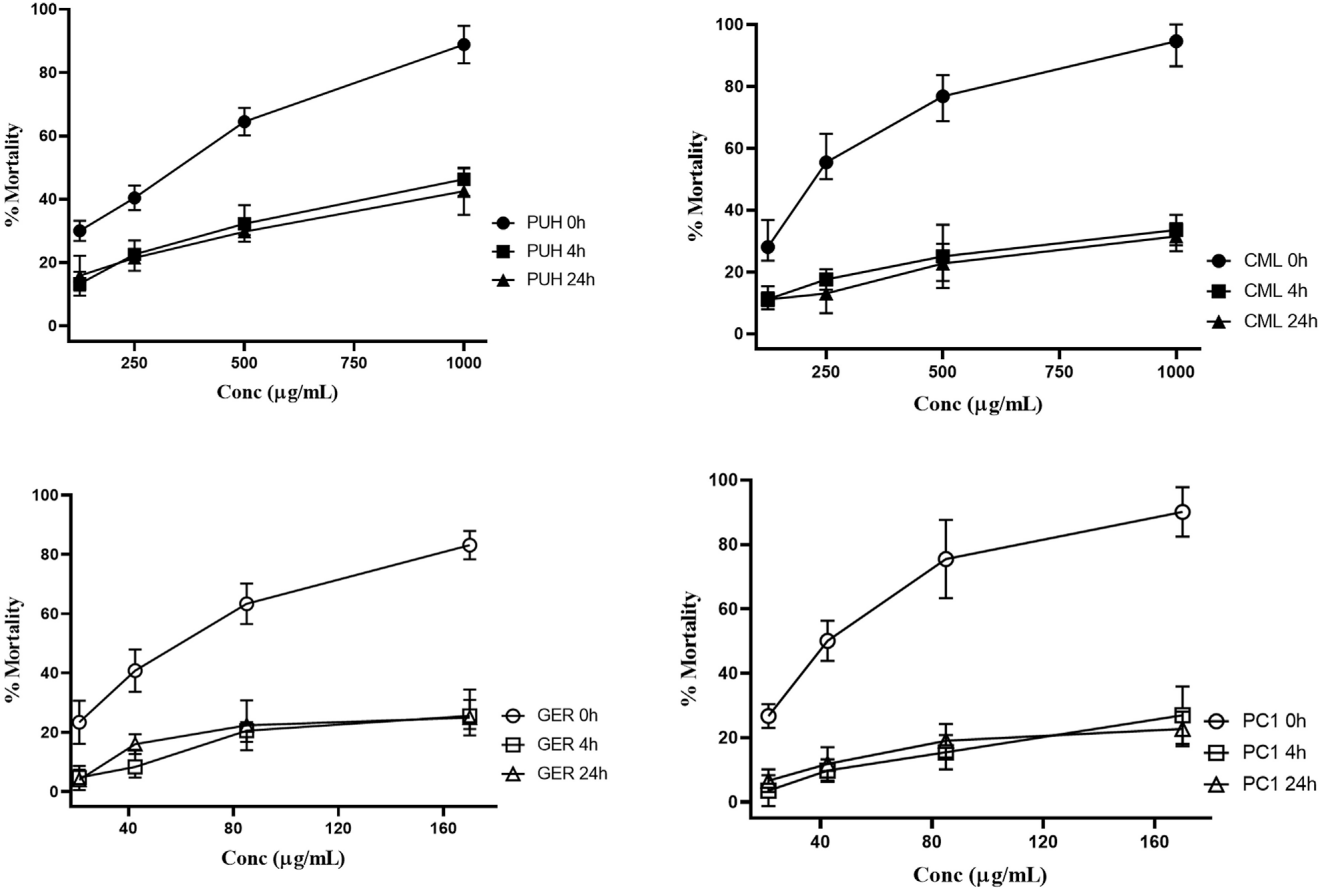
Relative anthelmintic activities of PUH **(A)**, CML **(B)**, geraniin **(C)**, and procyanidin C1 **(D)** without and after 4 and 24 h fermentation with human fecal suspension against *Trichuris trichiura* L1 after 24 h incubation at 37°C. PUH, *Phyllanthus urinaria* extract; CML, *C. mucronatum* extract; GER, geraniin; PC1, procyanidin C1. Bars represent mean ± SEM from three independent experiments with three technical replicates.

Tannin-enriched extracts from *P. urinaria* and *C. mucronatum* and two tannins (geraniin and procyanidin C1) were tested against the larvae of hookworm and *T. trichiura* isolated from human stool and against *C. elegans*. As displayed in Table 1, all test samples exerted lethal effects against the parasites. This appears to be the first set of data describing *in vitro* anthelmintic activities of tanniferous plant extracts against parasitic nematodes isolated from human fecal samples. The unfermented samples (0 h values) representing brief exposure of the samples to fecal matter without initiating sufficient metabolism exerted strong inhibition of the hookworms and whipworms (Table 1; Figures 3, 4). Mortality tests against these pathogens using the major compounds geraniin from *P. urinaria* and procyanidin C1 from *C. mucronatum* indicated strong activity, with LC_50_ values ranging from 49.6 (CI: 41.7–59.3) μg/mL to 63.5 (CI: 47.6–84.0) μg/mL, while fermentation of the extracts and compounds for 4 and 24 h in human fecal suspension led to varied decreases in the anthelmintic activity, as presented in Table 1.

**TABLE 1:** Summary of the anthelmintic activity of tannin-containing extracts PUH and CML, proanthocyanidin PC1, and ellagitannin GER against different nematode species. Samples at 0 h were not fermented, whereas 4 h and 24 h indicate fecal metabolism for 4 h and 24 h, respectively. LC_50_ values are calculated from triplicate determinations of n = 3 experiments and expressed with 95% confidence interval. ABZ: positive control albendazole.

Sample	Nematode LC_50_ (µg/mL) (95% CI)		
	Hookworm (L3)	*T*. *trichiura* (L1)	*C. elegans* (L4)
PUH 0 h	300.8 (245.1–374.8)	331.6 (290.3–382.5)	>2,000 μg/mL
CML 0 h	343.5 (267.5–445.4)	230.1 (198.9–271.2)	1468.1 (990.3–1,946.5)
PUH 4 h	852.0 (713.0–953.2)	(n.d.)	(n.d.)
CML 4 h	790.0 (616.1–985.9)	(n.d.)	(n.d.)
PUH 24 h	(n.d.)	(n.d.)	(n.d.)
CML 24 h	(n.d.)	(n.d.)	(n.d.)
LC_50_ (µM) (95% CI)			
GER 0 h	63.5 (47.6–84.0)	49.6 (41.7–59.7)	(n.d.)
PC1 0 h	60.9 (46.7–80.6)	50.4 (41.0–62.9)	(n.d.)
GER 4 h	(n.d.)	(n.d.)	(n.d.)
PC1 4 h	(n.d.)	(n.d.)	(n.d.)
GER 24 h	(n.d.)	(n.d.)	(n.d.)
PC1 24 h	(n.d.)	(n.d.)	(n.d.)
ABZ	22.3 (18.1–28.5)	34.3 (26.5–44.0)	na

(n.d.): LC_50_ could not be determined at tested concentrations; inhibition was not significant to achieve LC_50_. na, not tested in this organism.

PUH and CML retained some of their activity after fermentation, still causing mortality in 20%–30% of the nematodes. For GER and PC1, on the other hand, the mortality rate did not exceed 20% even at the highest concentration tested. The 4-h transit in the colon may be sufficient time for the metabolism of ingested tannins since much of the activity was lost after 4 h of fermentation. Although there was no significant variation in the effects between 4 h and 24 h for the test compounds, the extracts were more active at the 4 h than at the 24 h fermentation time against hookworms.

Unlike the two structurally distinct compounds (Figure 1) whose activity against the human intestinal parasites were similar, the activity of the two extracts, PUH and CML, varied against both organisms. Although the PUH extract (0 h) induced LC_50_ of 300.8 (CI: 245.1–374.8) μg/mL and 331.6 (CI: 290.3–382.5) μg/mL against hookworm and whipworm, respectively, the CML extract (0 h) yielded LC_50_ of 343.5 (CI: 267.5–445.4) μg/mL and 230.1 (CI: 198.9–271.2) μg/mL, respectively. The similar effects seen in the compounds could be attributed to the nonspecific astringent effects of these tannins, leading to a general inhibition of protein-related processes in the helminths (Luck et al., 1994; Spiegler et al., 2017), and this differed for the extracts which are composed of very varied phytoconstituents.

**FIGURE 5:**
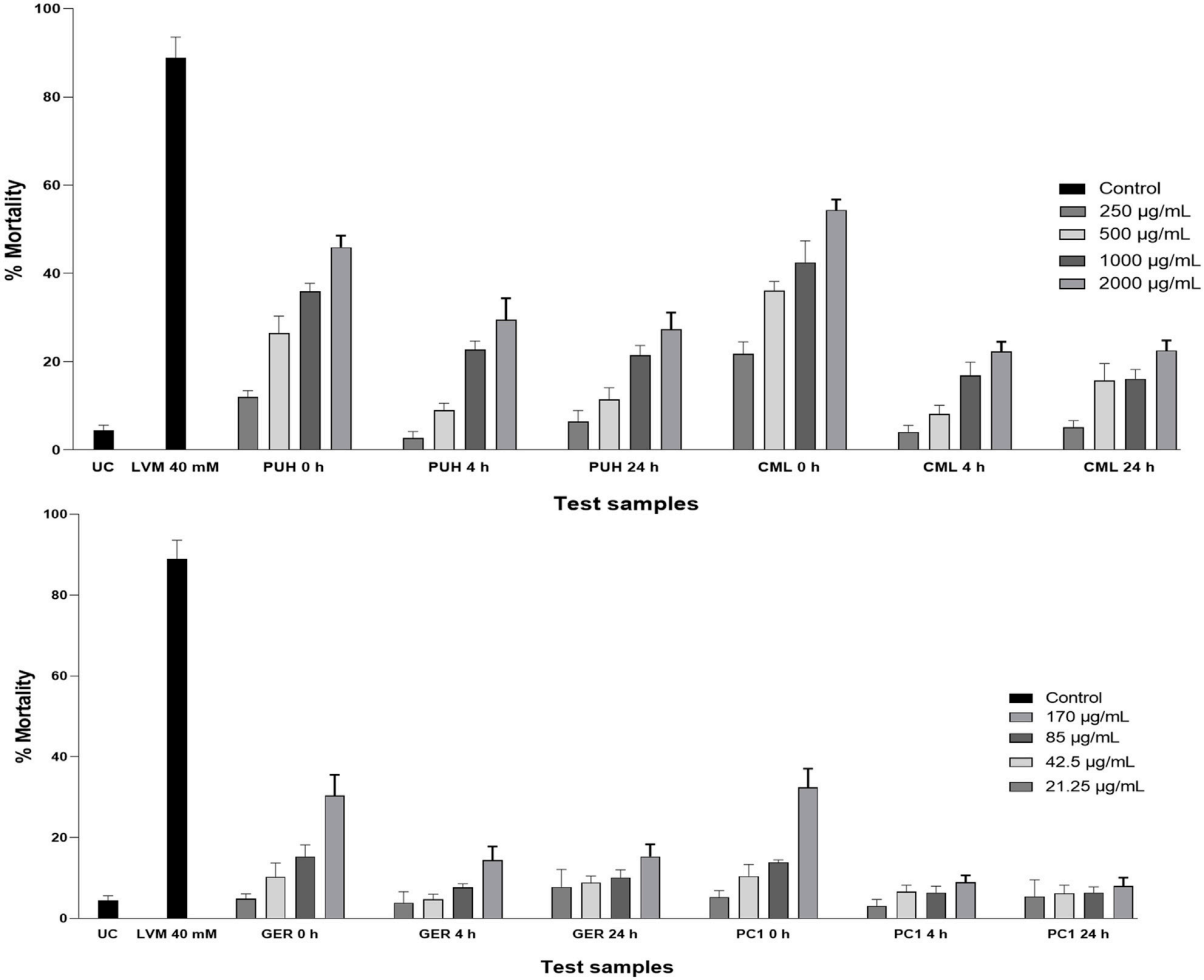
Relative anthelmintic activity of PUH and CML extracts **(A)** and geraniin and procyanidin C1 **(B)** without and after 4 and 24 h fermentation with human fecal suspension against *C. elegans* (wild-type N2 Bristol) after 72 h incubation at 20°C. UC, untreated control (M9 medium); LVM, positive control (levamisole HCl, 40 mM); PUH, *Phyllanthus urinaria* extract; CML, *C. mucronatum* extract; GER, geraniin; PC1, procyanidin C1. Bars represent mean ± SEM from three independent experiments with three technical replicates.

Treatment against stage-4 (L4) *C. elegans* larvae revealed similar effects in previous reports (Jato et al., 2023). In the unfermented state, the CML extract induced LC_50_ of 1,468.1 (CI: 990.3–1,946.5). The PUH extract and two compounds could not achieve inhibition that was sufficient to determine the LC_50_ values. All fermented samples (4 h and 24 h) had values lower than LC_50_ inhibition against this non-parasitic nematode (Table 1; Figure 5). The inhibitory activity of geraniin at 170 μg/mL (178.5 µM), however, decreased significantly from 30.4% ± 1.8% to 14.5% ± 1.5% when fermented for 4 h. A similar pattern was observed for PC1, for which the respective activity at 170 μg/mL (196.2 µM) against *C. elegans* larvae decreased from 32.4% ± 2.3% to 8.9% ± 0.9% after fermenting for 4 h.

Albendazole, which remains the drug of choice for both worm types, has been reported to be more active against hookworms than whipworms (Keiser and Utzinger, 2008). Within the current study, LC_50_ values for albendazole were in a very similar range, with 22.3 (CI: 18.1–28.5) µM and 34.3 (CI: 26.5–44.0) µM against hookworm and whipworm larvae, respectively.

## 4 Discussion

The anthelmintic potential of tannins has been extensively studied and considered an important area for anthelmintic drug development (Spiegler et al., 2017). Some studies have pointed to the bulky tannin structure and astringency as the properties responsible for such actions. These molecules (both ellagitannins and proanthocyanidins) have poor oral bioavailability and often reach the colon, where they are extensively metabolized by the gut microbiota into various smaller molecules whose therapeutic potential has not been fully explored (Blaut and Clavel, 2007). The two selected plants, *P. urinaria* and *C. mucronatum*, have considerable ethno-medicinal use as anthelmintic agents across Africa, and hence, it was essential to evaluate whether their fecal metabolites contribute to their anthelmintic action.

The inhibitory effects of the two tannins and their respective plant extracts significantly decreased against hookworm and whipworm larvae after fecal fermentation. Both geraniin and procyanidin C1 showed more pronounced anthelmintic effects against the parasites in the unfermented form (0 h group) than when they were metabolized (4 h and 24 h groups). A similar observation was made for the extracts, even though the decrease in anthelmintic activity was higher for the pure compounds. Although the components of the extract are often surrounded by a protective matrix, which may shield them from degradation by gut enzymes, individual compounds are often exposed, and hence, they are degraded easily. The extracts contain a wide array of other compounds whose metabolism may differ from the two isolated compounds. These other components, be they tannins or not, may still be available at high concentrations or yield different metabolites that inhibit the parasites, although to a lesser extent.

As previously indicated, the whipworm larvae appeared to be more sensitive to the test substances than hookworm larvae, even though hookworm is more prevalent in the study population (Humphries et al., 2012; Ahiadorme and Morhe, 2020). Most of the whipworm larvae were immotile within 24 h of treatment, which was more than what was observed in the hookworms. This is similar to investigations in animal parasites where the *T. vulpis* and *T. suis* larvae were more sensitive to PAC and ET treatment than hookworm and roundworm strains (Spiegler et al., 2016; Jato et al., 2023). This observation may present huge implications for parasite control and influence the dosage regimen for herbal remedies. Individuals with whipworm infection who are treated with these tannin-rich extracts may achieve a quicker decline in parasite burden than those infected with hookworms. This difference can inform the treatment regimen for mono- and poly-parasitism in the population, and in the case of hookworm-only-infection, multiple treatments with tannin-containing extracts may be required to achieve effective parasite control.

The investigation against *C. elegans* showed a similar pattern to the observations made in human intestinal nematodes. The compounds and extracts all produced significantly more inhibition in the unmetabolized form than in the fecal degraded state. Even though *C. elegans* is not a perfect model for parasitic nematodes due to genetic and metabolic disparities, it is a free-living, bacteria-feeding nematode and a well-established model organism for anthelmintic activity studies and remains the closest to parasitic nematodes (Geary and Thompson, 2001). It is, therefore, the preferred strain when comparing anthelmintic action in parasitic and non-parasitic nematodes. This organism has been reported to be less susceptible to anthelmintic agents compared to its parasitic counterparts (Ash et al., 1994; Knopp et al., 2008), and as was observed in this study, the extracts and compounds, even in their unfermented state, had low activity against *C. elegans* compared to that against the two parasitic worms (Figure 5).

### 4.1 Effects of metabolism on the anthelmintic action of tannins

Tannins generally exhibit non-specific activities against nematodes, owing to their bulky nature, which enhances complexation with proteins and other structural elements. Since tannin–protein interactions are essential for anthelmintic action, intact, non-metabolized tannins which retain their astringency may be desirable for the inhibition of intestinal parasites (Spiegler et al., 2017). These interactions appear to be non-specific and will be more pronounced for the bulky tannins than for their breakdown products (Luck et al., 1994; Stiernagle, 2006; Santos et al., 2019; Ahiadorme and Morhe, 2020). In this study, the intact, undegraded tannins and tannin-containing extracts produced significant anthelmintic activity compared to the fermented state. This suggests that orally administered tannins must pass the stomach undegraded to reach the intestines, where they are required for the inhibition of intestinal parasites.

Anthelmintic activities of tannins vary widely among structurally related or non-related molecules and among the different strains, species, and developmental stage of nematodes (Lans, 2006; Wimmersberger et al., 2013; Agyare et al., 2014). The variation in activities between geraniin and procyanidin C1 and the two extracts support these previous observations. Although both tannin groups may display similar inhibitory actions against larval migration, hydrolyzable tannins have been reported to have higher inhibition against egg-hatching (Burden and Hammet, 1976; Santos et al., 2019).

As previously reported, anthelmintic activities of hydrolyzable tannins are generally related to a molecular weight of 700–2,000 Da and are proportional to the degree of galloylation or number of hexahydroxydiphenyl moieties (Humphries et al., 2012; Spiegler et al., 2016). Since fecal metabolism breaks down these galloylated structures into low-molecular-weight molecules, this size- and galloylation-dependent action is lost and may explain the decline in the anthelmintic activity of geraniin and *P. urinaria* extract, as observed in this study. The smaller sized building blocks of procyanidins ((epi)catechins), for instance, have been reported to have little to no anthelmintic effects against intestinal nematodes of ruminants, *A. suum* of pigs, and *C. elegans* (Burden and Hammet, 1976; Geary and Thompson, 2001; Lans, 2006). Their galloylated forms, however, possess significant anthelmintic activity (Mukai et al., 2008; Williams et al., 2014a; Desrues et al., 2016), and this supports the idea that high-molecular weight PAC have better anthelmintic action, which could diminish due to degradation by intestinal flora (Williams et al., 2014b; Klongsiriwet et al., 2015; Quijada et al., 2015; Spiegler et al., 2017).

There is no sufficient data on the anthelmintic potential of tannin metabolites or degradation products, and hence, no direct link can be established between the individual metabolites and any residual anthelmintic action. The wide variations in anthelmintic activity of tannins are, therefore, highly dependent on physiological differences in nematode species and the stage of parasite development than on the products of tannin metabolism (Spencer et al., 1988; Hagerman et al., 1998). Since the bulky tannin molecule appears to be responsible for the anthelmintic action, the rate at which the molecules get degraded may be a predictor of overall anthelmintic potential. Geraniin, for instance, gets converted by fecal microflora to corilagin, brevifolin carboxylic acid, ellagic acid, and gallic acid within 1 h of anaerobic incubation and subsequently forms urolithins after 96 h (Ito et al., 2008). Although some of the initial products have been reported for anthelmintic action (Jato et al., 2023), the final products have not been reported for such activities. Four hours of fermentation appears to significantly lower the anthelmintic action of the test samples and suggest that intestinal transformation of this molecule may be quicker than 4 hours. Future studies could assess the rate of degradation and trend of metabolites produced from fermentation of both groups of tannins, and when fully characterized, the individual metabolites can be explored for nematicidal properties.

The results of this study and prevailing evidence seem to suggest that tannins exhibit direct effects on parasites rather than through their gut metabolites. This study also confirms that the extracts appear to have higher effects than the pure compounds and retain significant residual action after 4 h and 24 h incubation. This may be suggestive of synergistic or potentiation mechanisms exhibited by the multi-component extracts compared to single molecules. These findings may, hence, support the veterinary and ethno-medicinal applications of tannin-rich plants as fodder or extracts and not as purified compounds and lend support to polyherbal over mono-entity therapies.

### 4.2 Conclusion

This study revealed that geraniin and procyanidin C1 and the extracts of *P. urinaria* and *C. mucronatum* display significant anthelmintic activities against the larvae of human intestinal hookworms and whipworms and against the model organism *C. elegans.* These activities are, however, significantly decreased when these substances are fermented in human fecal matter for 4 h and 24 h. These findings suggest that tannins may have better anthelmintic action in their intact or unchanged form and validate the use of these two medicinal plants as anthelmintic agents in Africa. Studies in other stages of nematode life cycle could provide a complete profile on whether these fermentation by-products contribute to anthelmintic effects at all.

## Data Availability

The original contributions presented in the study are included in the article/Supplementary Material; further inquiries can be directed to the corresponding author.

## Abbreviations

CT, condensed tannin; CML, 50% hydroethanolic extract of *C. mucronatum* leaves; ET, ellagitannin; GER, geraniin; GIN, gastrointestinal nematode; HFS, human fecal suspension; HT, hydrolyzable tannin; PAC, proanthocyanidin; PC1, procyanidin C1; PUH, 70% hydro-acetonic extract of the aerial parts of *P. urinaria*; UC, untreated control.

## References

[B1] Agyare C. Asase A. Lechtenberg M. Niehues M. Deters A. Hensel A. (2009). An ethnopharmacological survey and *in vitro* confirmation of ethnopharmacological use of medicinal plants used for wound healing in Bosomtwi-Atwima-Kwanwoma area, Ghana. J. Ethnopharmacol. 125, 393–403. 10.1016/j.jep.2009.07.024 19635544

[B2] Agyare C. Spiegler V. Sarkodie H. Asase A. Liebau E. Hensel A. (2014). An ethnopharmacological survey and *in vitro* confirmation of the ethnopharmacological use of medicinal plants as anthelmintic remedies in the Ashanti region, in the central part of Ghana. J. Ethnopharmacol. 158, 255–263. 10.1016/j.jep.2014.10.029 25446638

[B3] Ahiadorme M. Morhe E. (2020). Soil transmitted helminth infections in Ghana: a ten year review. Pan Afr. Med. J. 35, 131–216. 10.11604/pamj.2020.35.131.21069 PMC7335259 32655745

[B4] Ash R. L. Orihel T. C. Savioli L. (1994). Bench aids for the diagnosis of intestinal parasites. Word Heal Organ 54, 548–571.

[B5] Banc R. Rusu M. E. Filip L. Popa D. S. (2023). The impact of ellagitannins and their metabolites through gut microbiome on the gut health and brain wellness within the gut–brain axis. Foods 12, 270. 10.3390/foods12020270 36673365 PMC9858309

[B6] Bharali R. Tabassum J. Azad M. (2003). Chemopreventive action of *Phyllanthus urinaria* Linn on DMBA-induced skin carcinogenesis in mice. Inidan J. Exp. Biol. 41, 1325–1328. 15332506

[B7] Bicker J. Petereit F. Hensel A. (2009). Proanthocyanidins and a phloroglucinol derivative from *Rumex acetosa* L. Fitoterapia 80, 483–495. 10.1016/j.fitote.2009.08.015 19695312

[B8] Blaut M. Clavel T. (2007). Metabolic diversity of the intestinal microbiota: implications for health and disease. J. Nutr. 137, 751S–755S. 10.1093/jn/137.3.751S 17311972

[B9] Burden D. J. Hammet N. C. (1976). A comparison of the infectivity of *Trichuris suis* ova embryonated by four different methods. Vet. Parasitol. 2, 307–311. 10.1016/0304-4017(76)90090-x

[B10] Cerdá B. Espín J. C. Parra S. Martínez P. Tomás-Barberán F. A. (2004). The potent *in vitro* antioxidant ellagitannins from pomegranate juice are metabolised into bioavailable but poor antioxidant hydroxy-6H-dibenzopyran-6-one derivatives by the colonic microflora of healthy humans. Eur. J. Nutr. 43, 205–220. 10.1007/s00394-004-0461-7 15309440

[B11] Cerdá B. Llorach R. Cerón J. J. Espín J. C. Tomás-Barberán F. A. (2003). Evaluation of the bioavailability and metabolism in the rat of punicalagin, an antioxidant polyphenol from pomegranate juice. Eur. J. Nutr. 42, 18–28. 10.1007/s00394-003-0396-4 12594538

[B12] Cerdá B. Tomás-Barberán F. A. Espín J. C. (2005). Metabolism of antioxidant and chemopreventive ellagitannins from strawberries, raspberries, walnuts, and oak-aged wine in humans: identification of biomarkers and individual variability. J. Agric. Food Chem. 53, 227–235. 10.1021/jf049144d 15656654

[B13] Charlier J. Vande F. Voort M. V. Van Meensel J. Lauwers L. Cauberghe V. (2015). ECONOHEALTH: placing helminth infections of livestock in an economic and social context. Vet. Parasitol. 15, 62–67. 10.1016/j.vetpar.2015.06.018 26159836

[B14] Desrues O. Fryganas C. Ropiak H. M. Mueller-Harvey I. Enemark H. L. Thamsborg S. M. (2016). Impact of chemical structure of flavanol monomers and condensed tannins on *in vitro* anthelmintic activity against bovine nematodes. Parasitology 143, 444–454. 10.1017/S0031182015001912 26888630 PMC4800716

[B16] Espín J. C. Larrosa M. García-Conesa M. T. Tomás-Barberán F. (2013). Biological significance of urolithins, the gut microbial ellagic acid-derived metabolites: the evidence so far. Evidence-based Complement. Altern. Med. 2013, 270418. 10.1155/2013/270418 PMC3679724 23781257

[B17] García-Villalba R. Giménez-Bastida J. A. Cortés-Martín A. Ávila-Gálvez M. Á. Tomás-Barberán F. A. Selma M. V. (2022). Urolithins: a comprehensive update on their metabolism, bioactivity, and associated gut microbiota. Mol. Nutr. Food Res. 66, e2101019. 10.1002/mnfr.202101019 35118817 PMC9787965

[B18] Geary T. G. (2012). Are new anthelmintics needed to eliminate human helminthiases? Curr. Opin. Infect. Dis. 25, 709–717. 10.1097/QCO.0b013e328359f04a 23041774

[B19] Geary T. G. Thompson D. P. (2001). *Caenorhabditis elegans*: how good a model for veterinary parasites? Vet. Parasitol. 101, 371–386. 10.1016/S0304-4017(01)00562-3 11707307

[B20] Gonthier M. P. Cheynier V. Donovan J. L. Manach C. Morand C. Mila I. (2003b). Microbial aromatic acid metabolites formed in the gut account for a major fraction of the polyphenols excreted in urine of rats fed red wine polyphenols. J. Nutr. 133, 461–467. 10.1093/jn/133.2.461 12566484

[B21] Gonthier M. P. Verny M. A. Besson C. Rémésy C. Scalbert A. (2003a). Chlorogenic acid bioavailability largely depends on its metabolism by the gut microflora in rats. J. Nutr. 133, 1853–1859. 10.1093/jn/133.6.1853 12771329

[B22] Greiffer L. Liebau E. Herrmann F. C. Spiegler V. (2022). Condensed tannins act as anthelmintics by increasing the rigidity of the nematode cuticle. Sci. Rep. 12, 18850–18913. 10.1038/s41598-022-23566-2 36344622 PMC9640668

[B23] Hagerman A. E. Riedl K. M. Jones G. A. Sovik K. N. Ritchard N. T. Hartzfeld P. W. (1998). High molecular weight plant polyphenolics (tannins) as biological antioxidants. J. Agric. Food Chem. 46, 1887–1892. 10.1021/jf970975b 29072454

[B25] Herrmann F. C. Spiegler V. (2019). *Caenorhabditis elegans* revisited by atomic force microscopy – ultra-structural changes of the cuticle, but not in the intestine after treatment with *Combretum mucronatum* extract. J. Struct. Biol. 208, 174–181. 10.1016/j.jsb.2019.08.013 31476367

[B26] Holt R. R. Lazarus S. A. Cameron S. M. Zhu Q. Y. Schramm D. D. Hammerstone J. F. (2002). Procyanidin dimer B2 [epicatechin-(4beta-8)-epicatechin] in human plasma after the consumption of a flavanol-rich cocoa. Am. J. Clin. Nutr. 76, 798–804. 10.1093/ajcn/76.4.798 12324293

[B28] Hoste H. Torres-Acosta J. F. J. Sandoval-Castro C. A. Mueller-Harvey I. Sotiraki S. Louvandini H. (2015). Tannin containing legumes as a model for nutraceuticals against digestive parasites in livestock. Vet. Parasitol. 212, 5–17. 10.1016/j.vetpar.2015.06.026 26190131

[B29] Hout S. Chea A. Bun S. Elias R. Gasquet M. Timon-David P. (2006). Screening of selected indigenous plants of Cambodia for antiplasmodial activity. J. Ethnopharmacol. 107, 12–18. 10.1016/j.jep.2006.01.028 16546336

[B31] Humphries D. Nguyen S. Boakye D. Wilson M. Cappello M. (2012). The promise and pitfalls of mass drug administration to control intestinal helminth infections. Curr. Opin. Infect. Dis. 25, 584–589. 10.1097/QCO.0b013e328357e4cf 22903231

[B32] Ilett K. F. Tee L. B. G. Reeves P. T. Minchin R. F. (1990). Metabolism of drugs and other xenobiotics in the gut lumen and wall. Pharmacol. Ther. 46, 67–93. 10.1016/0163-7258(90)90036-2 2181492

[B33] Ito H. Iguchi A. Hatano T. (2008). Identification of urinary and intestinal bacterial metabolites of ellagitannin geraniin in rats. J. Agric. Food Chem. 56, 393–400. 10.1021/jf0726942 18163562

[B34] Jato J. Orman E. Duah Boakye Y. Oppong Bekoe E. Oppong Bekoe S. Asare-Nkansah S. (2022). Anthelmintic agents from African medicinal plants: review and prospects. Evidence-based Complement. Altern. Med. 2022, 8023866. 10.1155/2022/8023866 PMC9825222 36624864

[B35] Jato J. Waindok P. Belga F. N. Orman E. Agyare C. Bekoe E. O. (2023). Anthelmintic activities of extract and ellagitannins from *Phyllanthus urinaria* against *Caenorhabditis elegans* and zoonotic or animal parasitic nematodes. Planta Med. 89 (13), 1215–1228. 10.1055/a-2117-9426 37459860

[B36] Kahle K. Huemmer W. Kempf M. Scheppach W. Erk T. Richling E. (2007). Polyphenols are intensively metabolized in the human gastrointestinal tract after apple juice consumption. J. Agric. Food Chem. 55, 10605–10614. 10.1021/jf071942r 18047284

[B37] Karonen M. Ahern J. R. Legroux L. Suvanto J. Engström M. T. Sinkkonen J. (2020). Ellagitannins inhibit the exsheathment of *Haemonchus contortus* and *Trichostrongylus colubriformis* larvae: the efficiency increases together with the molecular size. J. Agric. Food Chem. 68, 4176–4186. 10.1021/acs.jafc.9b06774 32181655 PMC7146859

[B38] Katz N. Chaves A. Pellegrino J. (1972). A simple device for quantitative stool thick-smear technique in *Schistosomiasis mansoni* . Rev. Inst. Med. Trop. Sao Paulo 14, 397–400. 4675644

[B39] Keiser J. Utzinger J. (2008). Efficacy of current drugs against soil-transmitted helminth infections: systematic review and meta-analysis. Clin. Corner 299, 1937–1948. 10.1001/jama.299.16.1937 18430913

[B40] Kisseih E. (2014). Phytochemical characterization and *in vitro* wound healing activity of leaf extracts from *Combretum mucronatum* . University of Muenster. 10.1016/j.jep.2015.06.008 26087235

[B41] Kisseih E. Lechtenberg M. Petereit F. Sendker J. Zacharski D. Brandt S. (2015). Phytochemical characterization and *in vitro* wound healing activity of leaf extracts from *Combretum mucronatum* Schum. and Thonn.: oligomeric procyanidins as strong inductors of cellular differentiation. J. Ethnopharmacol. 174, 628–636. 10.1016/j.jep.2015.06.008 26087235

[B43] Klongsiriwet C. Quijada J. Williams A. R. Mueller-Harvey I. Williamson E. M. Hoste H. (2015). Synergistic inhibition of *Haemonchus contortus* exsheathment by flavonoid monomers and condensed tannins. Int. J. Parasitol. Drugs Drug Resist 5, 127–134. 10.1016/j.ijpddr.2015.06.001 26199861 PMC4506970

[B44] Knopp S. Mgeni A. F. Khamis I. S. Steinmann P. Stothard J. R. Rollinson D. (2008). Diagnosis of soil-transmitted helminths in the era of preventive chemotherapy: effect of multiple stool sampling and use of different diagnostic techniques. PLoS Negl. Trop. Dis. 2, e331. 10.1371/journal.pntd.0000331 18982057 PMC2570799

[B45] Knopp S. Rinaldi L. Khamis I. S. Stothard J. R. Rollinson D. Maurelli M. P. (2009). A single FLOTAC is more sensitive than triplicate Kato-Katz for the diagnosis of low-intensity soil-transmitted helminth infections. Trans. R. Soc. Trop. Med. Hyg. 103, 347–354. 10.1016/j.trstmh.2008.11.013 19168197

[B46] Koné W. M. Vargas M. Keiser J. (2012). Anthelmintic activity of medicinal plants used in Côte d’Ivoire for treating parasitic diseases. Parasitol. Res. 110, 2351–2362. 10.1007/s00436-011-2771-z 22200959

[B47] Lans C. A. (2006). Ethnomedicines used in Trinidad and Tobago for urinary problems and diabetes mellitus. J. Ethnobiol. Ethnomed 2, 45–11. 10.1186/1746-4269-2-45 17040567 PMC1624823

[B48] Luck G. Liao H. Murray N. J. Grimmer H. R. Warminski E. E. Williamson M. P. (1994). Polyphenols, astringency and proline-rich proteins. Phytochemistry 37, 357–371. 10.1016/0031-9422(94)85061-5 7765619

[B49] Ludwig I. A. Mena P. Calani L. Borges G. Pereira-Caro G. Bresciani L. (2015). New insights into the bioavailability of red raspberry anthocyanins and ellagitannins. Free Radic. Biol. Med. 89, 758–769. 10.1016/j.freeradbiomed.2015.10.400 26475039

[B50] Marín L. Miguélez E. M. Villar C. J. Lombó F. (2015). Bioavailability of dietary polyphenols and gut microbiota metabolism: antimicrobial properties. Biomed. Res. Int. 2015, 905215. 10.1155/2015/905215 25802870 PMC4352739

[B51] Max R. A. Kimambo A. E. Kassuku A. A. A. Mtenga L. A. Buttery P. J. (2007). Effect of tanniniferous browse meal on nematode faecal egg counts and internal parasite burdens in sheep and goats. South Afr. J. Anim. Sci. 37, 97–106. 10.4314/sajas.v37i2.4033

[B52] Min B. R. Barry T. N. Attwood G. T. McNabb W. (2003). The effect of condensed tannins on the nutrition and health of ruminants fed fresh temperate forages: a review. Anim. Feed Sci. Technol. 106, 3–19. 10.1016/S0377-8401(03)00041-5

[B53] Mukai D. Matsuda N. Yoshioka Y. Sato M. Yamasaki T. (2008). Potential anthelmintics: polyphenols from the tea plant *Camellia sinensis* L. are lethally toxic to *Caenorhabditis elegans* . J. Nat. Med. 62, 155–159. 10.1007/s11418-007-0201-4 18404315

[B54] Ndjonka D. Abladam E. D. Djafsia B. Ajonina-Ekoti I. Achukwi M. D. Liebau E. (2013). Anthelmintic activity of phenolic acids from the axlewood tree *Anogeissus leiocarpus* on the filarial nematode *Onchocerca ochengi* and drug-resistant strains of the free-living nematode *Caenorhabditis elegans* . J. Helminthol. 88, 481–488. 10.1017/S0022149X1300045X 23768773

[B55] Orman E. Oppong B. S. Asare-Nkansah S. Kralisch I. Jato J. Spiegler V. (2023). Towards the development of analytical monograph specifications for the quality assessment of the medicinal plant *Phyllanthus urinaria* . Phytochemistry 215, 113854. 10.1016/j.phytochem.2023.113854 37716546

[B56] Ou K. Gu L. (2014). Absorption and metabolism of proanthocyanidins. J. Funct. Foods 7, 43–53. 10.1016/j.jff.2013.08.004

[B57] Piwowarski J. P. Granica S. Stefańska J. Kiss A. K. (2016). Differences in metabolism of ellagitannins by human gut microbiota *ex vivo* cultures. J. Nat. Prod. 79, 3022–3030. 10.1021/acs.jnatprod.6b00602 28006907

[B58] Quijada J. Fryganas C. Ropiak H. M. Ramsay A. Mueller-Harvey I. Hoste H. (2015). Anthelmintic activities against *Haemonchus contortus* or *Trichostrongylus colubriformis* from small ruminants are influenced by structural features of condensed tannins. J. Agric. Food Chem. 63, 6346–6354. 10.1021/acs.jafc.5b00831 26066999

[B59] Rios L. Y. Gonthier M. P. Rémésy C. Mila I. Lapierre C. Lazarus S. A. (2003). Chocolate intake increases urinary excretion of polyphenol-derived phenolic acids in healthy human subjects. Am. J. Clin. Nutr. 77, 912–918. 10.1093/ajcn/77.4.912 12663291

[B60] Rose V. H. Morgan E. R. Hertzberg H. (2020). Increasing importance of anthelmintic resistance in European livestock: creation and meta-analysis of an open database. Parasite 27, 1–16. 10.1051/parasite/2020062 33277891 PMC7718593

[B61] Sano A. Yamakoshi J. Tokutake S. Tobe K. Kubota Y. Kikuchi M. (2003). Procyanidin B1 is detected in human serum after intake of proanthocyanidin-rich grape seed extract. Biosci. Biotechnol. Biochem. 67, 1140–1143. 10.1271/bbb.67.1140 12834296

[B62] Santos F. O. Cerqueira A. P. M. Branco A. José Moreira Batatinha M. Borges Botura M. (2019). Anthelmintic activity of plants against gastrointestinal nematodes of goats: a review. Parasitology 146, 1233–1246. 10.1017/S0031182019000672 31104640

[B63] Satyan K. S. Prakash A. Singh R. P. Srivastava R. S. (1995). Phthalic acid bis-ester and other phytoconstituents of *Phyllanthus urinaria* . Planta 61, 293–294. 10.1055/s-2006-958083 17238083

[B64] Seeram N. P. Henning S. M. Zhang Y. Suchard M. Li Z. Heber D. (2006). Pomegranate juice ellagitannin metabolites are present in human plasma and some persist in urine for up to 48 hours. J. Nutr. 136, 2481–2485. 10.1093/jn/136.10.2481 16988113

[B65] Spencer C. M. Cai Y. Martin R. Gaffney S. H. Goulding P. N. Magnolato D. (1988). Polyphenol complexation-some thoughts and observations. Phytochemistry 27, 2397–2409. 10.1016/0031-9422(88)87004-3

[B66] Spiegler V. (2016). Anthelmintic activity of procyanidins from West African plants - from traditional medicine to phytochemistry and molecular investigations. Westfälischen Wilhelms-Universität Münster.

[B67] Spiegler V. (2020). Anthelmintic A-type procyanidins and further characterization of the phenolic composition of a root extract from *Paullinia pinnata* . Molecules 25, 2287. 10.3390/molecules25102287 32414042 PMC7287971

[B68] Spiegler V. Liebau E. Hensel A. (2017). Medicinal plant extracts and plant-derived polyphenols with anthelmintic activity against intestinal nematodes. Nat. Prod. Rep. 34, 627–643. 10.1039/c6np00126b 28426037

[B69] Spiegler V. Liebau E. Peppler C. Raue K. Werne S. Strube C. (2016). A hydroalcoholic extract from *Paullinia pinnata* L. roots exerts anthelmintic activity against free-living and parasitic nematodes. Planta Med. 82, 1173–1179. 10.1055/s-0042-108209 27286336

[B70] Spiegler V. Sendker J. Petereit F. Liebau E. Hensel A. (2015). Bioassay-guided fractionation of a leaf extract from *Combretum mucronatum* with anthelmintic activity: oligomeric procyanidins as the active principle. Molecules 20, 14810–14832. 10.3390/molecules200814810 26287140 PMC6332176

[B71] Stiernagle T. (2006). Maintenance of *C. elegans* . Wormb Ed. C. elegans Res. Community, Wormb, 1–11. 10.1895/wormbook.1.101.1 PMC4781397 18050451

[B72] Symma N. Sendker J. Petereit F. Hensel A. (2020). Multistep analysis of diol-LC-ESI-HRMS data reveals proanthocyanidin composition of complex plant extracts (PAComics). J. Agric. Food Chem. 68, 8040–8049. 10.1021/acs.jafc.0c02826 32633530

[B73] Tao W. Zhang Y. Shen X. Cao Y. Shi J. Ye X. (2019). Rethinking the mechanism of the health benefits of proanthocyanidins: absorption, metabolism, and interaction with gut microbiota. Compr. Rev. Food Sci. Food Saf. 18, 971–985. 10.1111/1541-4337.12444 33336996

[B74] WHO (2020). Ending the neglect to attain the sustainable development goals: a road map for neglected tropical diseases 2021–2030. Word Heal Organ 196.

[B75] WHO (2022). WHO Factsheet: soil-transmitted helminth infections. World Heal Organ, 1–5.

[B76] Williams A. R. Fryganas C. Ramsay A. Mueller-Harvey I. Thamsborg S. M. (2014b). Direct anthelmintic effects of condensed tannins from diverse plant sources against *Ascaris suum* . PLoS One 9, e97053. 10.1371/journal.pone.0097053 24810761 PMC4014605

[B77] Williams A. R. Ropiak H. M. Fryganas C. Desrues O. Mueller-Harvey I. Thamsborg S. M. (2014a). Assessment of the anthelmintic activity of medicinal plant extracts and purified condensed tannins against free-living and parasitic stages of *Oesophagostomum dentatum* . Parasites Vectors 7, 518–612. 10.1186/s13071-014-0518-2 25406417 PMC4240858

[B78] Wimmersberger D. Tritten L. Keiser J. (2013). Development of an *in vitro* drug sensitivity assay for *Trichuris muris* first-stage larvae. Parasites Vectors 6, 42–48. 10.1186/1756-3305-6-42 23433224 PMC3606346

[B79] Wiseman H. Casey K. Bowey E. A. Duffy R. Davies M. Rowland I. R. (2004). Influence of 10 wk of soy consumption on plasma concentrations and excretion of isoflavonoids and on gut microflora metabolism in healthy adults. Am. J. Clin. Nutr. 80, 692–699. 10.1093/ajcn/80.3.692 15321810

[B81] Zumdick S. Deters A. Hensel A. (2012). *In vitro* intestinal transport of oligomeric procyanidins (DP 2 to 4) across monolayers of Caco-2 cells. Fitoterapia 83, 1210–1217. 10.1016/j.fitote.2012.06.013 22776719

