# Supplementary material for: Influence of fecal fermentation on the anthelmintic activity of proanthocyanidins and ellagitannins against human intestinal nematodes and Caenorhabditis elegans
Source: Front Pharmacol. 2024 Jul 22;15:1390500. doi: 10.3389/fphar.2024.1390500 (PMC11298482; doi:10.3389/fphar.2024.1390500)
Supplement: Supplementary file 1 [file DataSheet1.docx]

***Supplementary Material***

**Supplementary Table 1**: Yield of fractions SF1-SF14 obtained from Sephadex LH-20 column chromatography of 10 g ethyl acetate fraction of *T. platyphyllos*.

| **Fraction** | **Fraction No [10 mL]** | **Approx. bulked volume [mL]** | **Yield weight**  **[g]** | **Yield**  **[% (*w*/*w*)]** |
| --- | --- | --- | --- | --- |
| SF1 | 1-14 | 140 | 0.1 | 1.0 |
| SF2 | 15-22 | 80 | 0.4 | 4.0 |
| SF3 | 23-32 | 100 | 0.8 | 8.3 |
| SF4 | 33-38 | 60 | 0.6 | 5.5 |
| SF5 | 39-59 | 210 | 1.7 | 17.3 |
| SF6 | 60-74 | 150 | 0.4 | 3.8 |
| SF7 | 75-90 | 160 | 0.4 | 4.4 |
| SF8 | 91-119 | 290 | 0.5 | 5.1 |
| SF9 | 120-260 | 1410 | 0.8 | 8.0 |
| SF10 | 261-338 | 780 | 0.2 | 2.4 |
| SF11 | 339-480 | 1420 | 0.3 | 2.6 |
| SF12 | 481-560 | 800 | 0.2 | 2.4 |
| SF13 | 561-600 | 400 | 0.3 | 2.7 |
| SF14 | 601-650 | 500 | 0.3 | 3.4 |
| **Total** | | | 7.1 | 71.0 |

**Supplementary Table 2:** Yield of subfractions SF7M1-8 and SF8M1-6 from MCI Gel™ CHP-20P column chromatography of SF7 (**440 mg)** and SF8 (**512 mg)**

| **Fraction** | **Fraction No. [10 mL]** | **Approx. bulked volume [mL]** | **Yield**  **[mg]** | **Yield**  **[% (*w*/*w*)]** |
| --- | --- | --- | --- | --- |
| SF7M1 | 1-39 | 468 | 4 | 0.9 |
| SF7M2 | 40-51 | 144 | 28 | 6.4 |
| SF7M3 | 52-58 | 84 | 198 | 45.0 |
| SF7M4 | 59-69 | 132 | 59 | 13.4 |
| SF7M5 | 70-78 | 108 | 17 | 3.9 |
| SF7M6 | 79-93 | 180 | 22 | 5.0 |
| SF7M7 | 94-101 | 96 | 18.5 | 4.2 |
| SF7M8 | 102-160 | 708 | 21 | 4.8 |
| **Total** | | | **367.5** | **83.6** |
| SF8M1 | 1-48 | 576 | 5.7 | 1.1 |
| SF8M2 | 49-67 | 228 | 288 | 56.2 |
| SF8M3 | 68-71 | 48 | 18.5 | 3.6 |
| SF8M4 | 72-75 | 48 | 15 | 2.9 |
| SF8M5 | 76-85 | 120 | 48 | 9.4 |
| SF8M6 | 86-160 | 900 | 32 | 6.3 |
| **Total** | | | **407.2** | **79.5** |

**Supplementary Table 3**: Yield of fractions i-v and PC1 from MCI gel chromatography of SF8M2. Yield [% (*w*/*w*)] is related to the starting amount (288 mg) of SF8M2.

| **Fraction** | **Yield [mg]** | **Yield [% (*w*/*w*)]** |
| --- | --- | --- |
| i | 2 | 0.7 |
| ii | 25 | 8.7 |
| iii | 25 | 8.7 |
| iv (PC1) | 94 | 32.6 |
| v | 96 | 33.3 |
| **Total** | **242** | **84.0** |

**
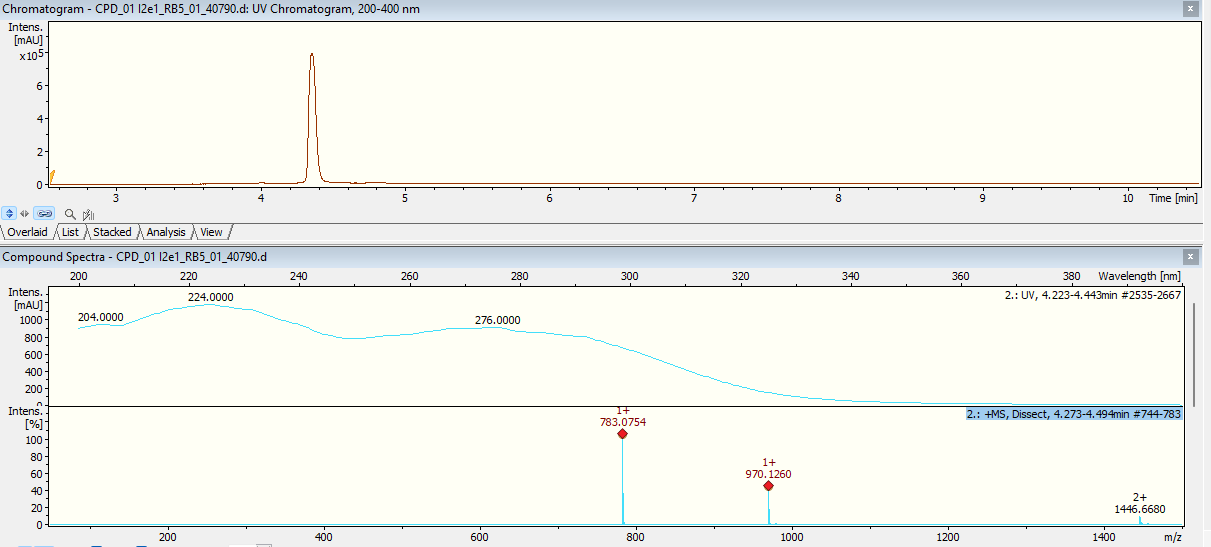
**

**Supplementary Figure 1:** UPLC-ESI-QTOF-MS spectra and UV chromatogram of geraniin (**1**)


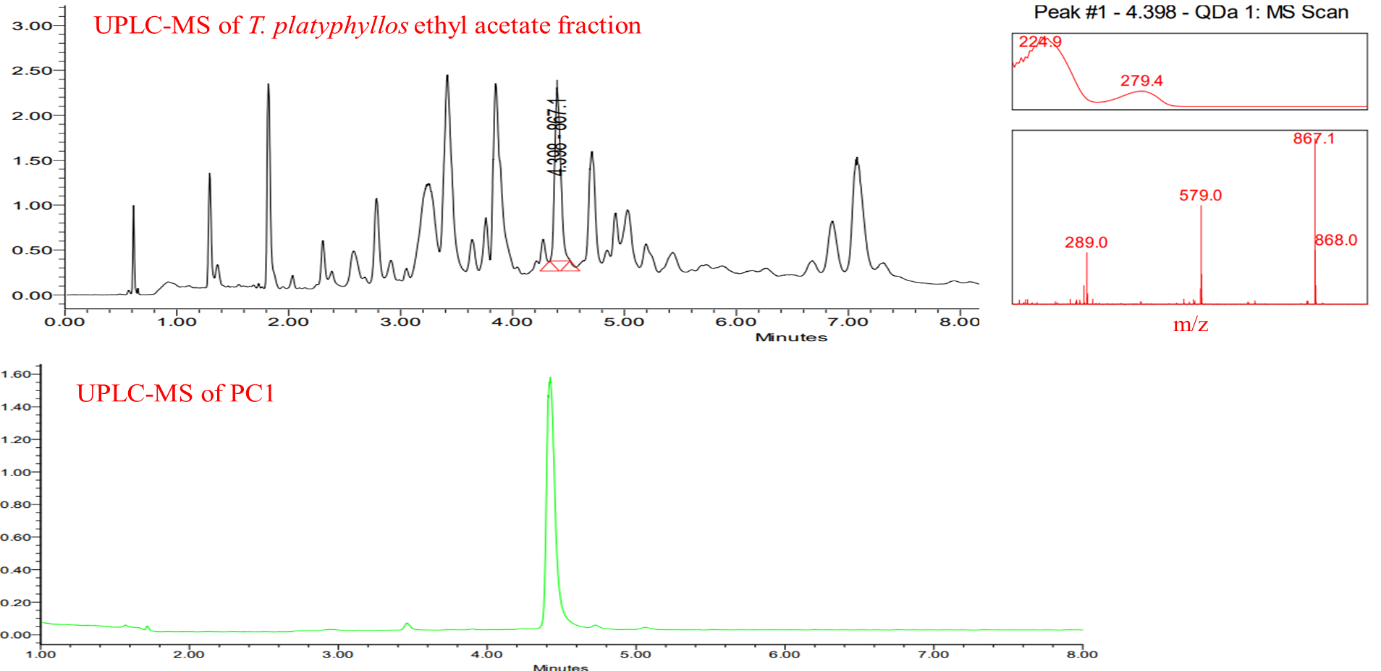


**Supplementary Figure 2:** UV chromatogram of *T. platyphyllos* ethyl acetate fraction (insert: MS of PC1 in the fraction) and PC1 after isolation.


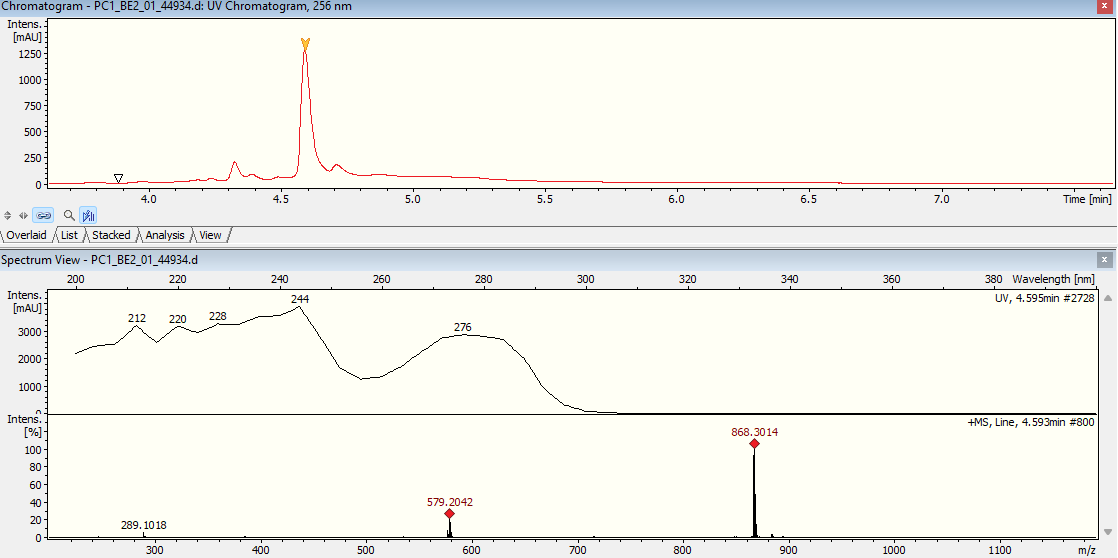


**Supplementary Figure 3:** UPLC-ESI-QTOF-MS Spectra and UV chromatogram of PC1.
